# Loss of a chloroplast encoded function could influence species range in kelp

**DOI:** 10.1002/ece3.5428

**Published:** 2019-07-09

**Authors:** Shivani Rana, Klaus Valentin, Inka Bartsch, Gernot Glöckner

**Affiliations:** ^1^ Medical Faculty, Institute of Biochemistry I University of Cologne Cologne Germany; ^2^ Alfred‐Wegener‐Institute, Helmholtz Center for Marine and Polar Research Bremerhaven Germany

**Keywords:** chloroplast genome analysis, habitat range, kelp, multinucleotide substitutions

## Abstract

Kelps are important providers and constituents of marine ecological niches, the coastal kelp forests. Kelp species have differing distribution ranges, but mainly thrive in temperate and arctic regions. Although the principal factors determining biogeographic distribution ranges are known, genomics could provide additional answers to this question.

We sequenced DNA from two *Laminaria* species with contrasting distribution ranges, *Laminaria digitata* and *Laminaria solidungula*. *Laminaria digitata* is found in the Northern Atlantic with a southern boundary in Brittany (France) or Massachusetts (USA) and a northern boundary in the Arctic, whereas *L. solidungula* is endemic to the Arctic only. From the raw reads of DNA, we reconstructed both chloroplast genomes and annotated them. A concatenated data set of all available brown algae chloroplast sequences was used for the calculation of a robust phylogeny, and sequence variations were analyzed.

The two *Laminaria* chloroplast genomes are collinear to previously analyzed kelp chloroplast genomes with important exceptions. Rearrangements at the inverted repeat regions led to the pseudogenization of *ycf*37 in *L. solidungula*, a gene possibly required under high light conditions. This defunct gene might be one of the reasons why the habitat range of *L. solidungula* is restricted to lowlight sublittoral sites in the Arctic. The inheritance pattern of single nucleotide polymorphisms suggests incomplete lineage sorting of chloroplast genomes in kelp species.

Our analysis of kelp chloroplast genomes shows that not only evolutionary information could be gleaned from sequence data. Concomitantly, those sequences can also tell us something about the ecological conditions which are required for species well‐being.

## 1 INTRODUCTION

Brown algae (Ochrophyta) have complex chloroplasts, that is, these organelles are surrounded by four membranes. Evolutionary, this has been explained by the occurrence of a secondary endosymbiosis, whereby a red alga was engulfed by a eukaryote host (Yoon, Hackett, Pinto, & Bhattacharya, 2002). Over time, the red alga was integrated into the host metabolism, thereby losing its complete nuclear genome. Kelps (Laminariales, Phaeophyceae) are large multicellular, highly differentiated marine brown algae. They can form huge coastal forests, which provide a habitat for microbes, animals, and other algae (Steneck et al., 2002). Thus, they construct an ecological niche dependent on their presence. Kelp forests are thriving along all temperate to polar rocky coastlines, but some forests also occur in deeper depth below the thermocline in tropical regions (Graham, Kinlan, Druehl, Garske, & Banks, 2007). Habitat ranges of different kelp species can overlap so that they can be present in a common forest. *Laminaria* species are found in the northern and southern Atlantic and northern Pacific but are not present in the southern Pacific and Antarctica (Lüning, 1990). *Laminaria digitata* thrives in the Northern Atlantic with a southern distribution boundary in Brittany (France) or Massachusetts (USA) and a northern limit in the Arctic whereas *L. solidungula* is restricted to the Arctic Ocean and often thrives at lower depths. Here, we wanted to analyze whether these contrasting distribution patterns might also find a reflection in their genomes.

Only a handful of brown algal nuclear genomes have so far been deciphered, namely *Ectocarpus siliculosus* (Cock et al., 2010), *Saccharina japonica* (Ye et al., 2015), and *Cladosiphon okamuranus* (Nishitsuji, Arimoto, & Iwai, 2016), *S. japonica* being the sole member of kelp species. Thus, it is currently not possible to comparatively examine complete nuclear genomes of kelp species for evolutionary changes and adaptations.

Chloroplast genomes generally have a quadripartite structure with a small and a large single copy region separated by inverted or direct repeats harboring at least the small and large ribosomal RNA subunits but exceptions are also known (Glöckner, Rosenthal, & Valentin, 2000).

The first completely deciphered and annotated chloroplast genome of a kelp species was that of *Saccharina japonica* (Wang et al., 2013). Two further kelp chloroplast genomes have also been published (Zhang, Wang, Liu, Wang, Chi, et al., 2015a; Zhang, Wang, Liu, Wang, Wang, et al., 2015b), resulting in only three available kelp chloroplast genomes so far. All the three kelp chloroplast genomes are conventionally quadripartite with inverted repeats restricted to the rRNA genes together with a few tRNA genes. Additionally, all three chloroplast genomes were collinear. We here present chloroplast genome data on two *Laminaria* species (*L. digitata* and *L. solidungula*) and compare all five chloroplast genomes. Our analysis reveals general trends of chloroplast genome evolution within kelp species.

## 2 MATERIAL AND METHODS

### 2.1 Algal material

Clonal male gametophytes of *Laminaria digitata* (AWI culture number 3157), originally isolated from Helgoland (North Sea), were cultivated at 8–15°C in sterilized filtered sea water under red light to avoid differentiation and to generate enough vegetative biomass for DNA extraction. Before DNA extraction, the gametophytes were washed three to six times with sterilized filtered seawater every second day to reduce the amount of bacteria in the culture.

Further isolates for the analysis of population differences came from Connecticut, USA (AWI culture number 3380), and Halifax, Canada (AWI culture number 3259), and non clonal vegetative gametophyte material (mixture of both sexes) which had been derived from spores collected in September 2018 at Roscoff and Quiberon (France) were used for DNA extraction and PCR and sequencing of chloroplast regions.

Sporophytes of *L. solidungula* were initiated from gametophytes (AWI culture number 3130, originally isolated from Kongsfjorden, Spitsbergen). After fertilization of the gametophytes in short day lengths (5:19 hr LD) at 0°C, they were transferred into 16:8 hr LD conditions, 5°C and a photon fluence rate of 40 µmol m^‐2^ s^‐1^ for further cultivation. Resulting sporophytes were sampled for DNA extraction when they had a size of approx. 5 cm. Gametophytes were sent to Cologne under cooled conditions within a working day before extraction. The sporophytes were cleaned with tissue paper and shock‐frozen in liquid N_2_ before freeze‐drying and extraction.

### 2.2 DNA extraction

After grinding, the tissue under liquid nitrogen DNA of *L. digitata* gametophytes was extracted from freshly drained material according to Doyle and Doyle modified cetyl trimethyl ammonium bromide method (CTAB; Doyle & Doyle 1990). The material from the freeze‐dried sample *of L. solidungula* was submitted to the same extraction method.

### 2.3 Sequencing, assembly, and chloroplast sequence extraction

Total DNA (5 µg) was converted to an Illumina sequencing library and analyzed on an Illumina Hiseq machine. Trimming and further processing were done with the Illumina software suit. Assembly was performed with abyss‐pe (Simpson et al., 2009) using kmers 40, 45, and 55. These assemblies were searched for similarity to the *S. japonica* chloroplast nucleotide sequence (JQ405663). Resulting contigs were used to reconstruct the complete chloroplast genomes by closing gaps with Gapfiller (Boetzer & Pirovano 2012).

PCR on *L. digitata* isolates was done with forward primer TTCATCAATAAATAAAAGACCACCCATTGC at position 75,636 to 75,665 and reverse primer TTCATCAATAAATAAAAGACCACCCATTGC at position 76,426 to 76,455. The resulting PCR products were ligated into pGem‐T Easy vectors. To be able to discern between polymerase errors and true SNPs, three clones from each ligation were sequenced.

### 2.4 Phylogenetic analysis

The chloroplast coding sequences of both *Laminaria* species were identified by blasting the CDS from *S. japonica* against the respective chloroplast sequences. Nucleotide sequences of the coding sequences were extracted and aligned gene‐wise using muscle (Edgar, 2004). The single alignments were inspected by eye and corrected, if needed. Concatenation of all single alignments was done with SCaFoS (Roure, Rodriguez‐Ezpeleta, & Philippe, 2007). The concatenated data set was used in a maximum‐likelihood approach for phylogenetic reconstruction with a discrete gamma distribution and with 1,000 bootstrap replications in MEGA6 (Tamura, Stecher, Peterson, Filipski, & Kumar, 2013).

### 2.5 Chloroplast genome analysis

Collinearity of the assembled kelp chloroplast genomes was tested with the nucmer tool of mummer (Kurtz, Phillippy, & Delcher, 2004), and a global alignment was done with MAFFT (Katoh & Standley 2013). The *Laminaria* chloroplast genomes were annotated using the available kelp chloroplast annotation as a BLAST query. Additionally, we detected tRNAs with the help of tRNA‐scan‐SE (Lowe & Eddy 1997) by searching all five kelp genomes using the organelle tRNA detection method. SNPs and small insertions/deletions can best be defined using software developed for the analysis of allelic differences in diploid eukaryote genomes. The raw sequence reads from *L. digitata* and *L. solidungula* were mapped to the *S. japonica* chloroplast genome as a reference. The *Costaria costata* and *Undaria pinnatifida* chloroplast genomes were downloaded from NCBI, and artificial raw reads were produced using the ArtificialFastqGenerator (Frampton & Houlston 2012). The reads of all chloroplast genomes were mapped to the reference genome using bowtie2 (Langmead & Salzberg 2012) resulting in a sorted bam file. The sequence variants were analyzed with The Genome Analysis Toolkit (Van der Auwera et al., 2013) and the resulting SNP library manually inspected for consistency.

## 3 RESULTS

### 3.1 The chloroplast genomes of *L. digitata* and *L. solidungula*

The sequencing total DNA yielded 179 million reads for *L. digitata* and 150 million reads for *L. solidungula* amounting to 12.3 and 11.3 gigabases, respectively. After assembly of all reads, we extracted the chloroplast contigs from the total assembly using the *Ectocarpus siliculosus* chloroplast coding sequences as a bait. Since the coverage of the chloroplast genomes is much higher than that of the nuclear genomes (estimated ~3,000× each for *L. solidungula* and for *L. digitata*), the assembly of so many reads results in a very fragmented chloroplast genome. Thus, the extracted chloroplast contigs were extended, scaffolded and the gaps between them were filled by using the original raw read information with the help of Gapfiller (Boetzer & Pirovano 2012). Extensions into the inverted repeats from both sides of the final single contig of each *Laminaria* species indicated completeness of the chloroplast genomes. We annotated the genomes using the available annotations for the other three kelp genomes and included de novo detection of tRNAs. With this approach, we defined 139 coding sequences each in the genomes and 29 (*L. digitata*) and 30 (*L. solidungula*) tRNAs together with three rRNA species (16S, 23S, and 5S) located in the inverted repeats. Since the number of tRNAs thus seems to differ between the chloroplast genomes of kelp species, we further analyzed, which tRNAs were affected by potential evolutionary processes. In total, we defined 36 tRNA locations on the chloroplast genomes of which 27 are located on the same position in all five kelp chloroplast genomes (Table A1). Of the remaining nine tRNAs, seven are present in only one species, one can be found in two species, and the remaining one is missing in *C. costata* only. Interestingly, six of the seven orphan tRNAs and the tRNA occurring in two genomes are predicted to contain type II introns.

### 3.2 The phylogeny of kelp genomes

**Figure 1:**
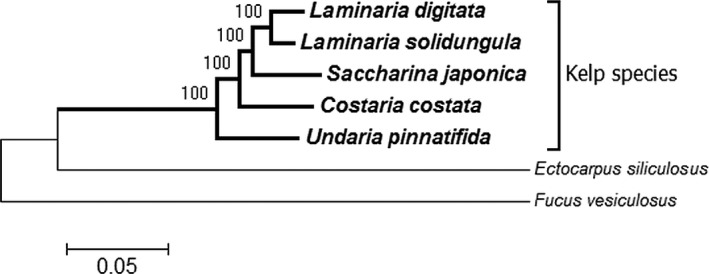
Phylogeny of Laminariales species (kelp) in comparison with other brown algae with completely sequenced chloroplast genomes. The tree was rooted with *Ectocarpus siliculosus* and *Fucus vesiculosus*. The evolutionary history was inferred by using the maximum‐likelihood method based on the general time reversible model (Nei & Kumar 2002; Tamura et al., 2012) with 1,000 bootstrap replications. The tree with the highest log likelihood (−249454.9341) is shown. The initial tree for the heuristic search was obtained by applying the neighbor‐joining method to a matrix of pairwise distances estimated using the maximum composite likelihood (MCL) approach. A discrete Gamma distribution was used to model evolutionary rate differences among sites (five categories (+*G*, parameter = 0.2099)). The rate variation model allowed for some sites to be evolutionarily invariable ([+*I*], 0.0000% sites). The tree is drawn to scale, with branch lengths measured in the number of substitutions per site. There were a total of 96,570 positions in the final dataset. Evolutionary analyses were conducted in MEGA6 (Tamura et al., 2013)

To be able to trace back the evolution of kelp species, we needed a robust phylogeny of the species analyzed. Thus, we extracted all coding sequences of the chloroplast genomes from *Undaria pinnatifida* (Zhang, Wang, Liu, Wang, Chi, et al., 2015a), *Costaria costata (*Zhang, Wang, Liu, Wang, Wang, et al., 2015b *)*, *Saccharina japonica (*Wang et al., 2013 *)*, the two *Laminaria* species analyzed here, and *Ectocarpus siliculosus* and *Fucus vesiculosus* (Le Corguille et al., 2009). All these chloroplast genomes had 137 coding sequences in common, the two open reading frames (ORFs) with undefined functions being restricted to kelp species. After alignment of the coding sequences of the respective individual genes, we concatenated these to yield a combined alignment of 96,570 bases. For the phylogenetic analysis, we used *E. siliculosus* and *F. vesiculosus* as outgroups. A model test indicated that the GTR + Gamma model would be best fitting for the data. Using this model with 1,000 bootstrap replications, we generated a phylogeny of the kelp species (Figure 1). Clearly, the *Laminaria* species group together, and the bootstrap values of the whole kelp tree indicate that the phylogenetic relationships of the species are well resolved. Sequence variations not following the species tree were also observed (see below) but the phylogenetic signal over the whole plastid genomes seems to be strong enough to be not influenced by them. This phylogeny was then the basis for further analysis of the observable trends in kelp chloroplast genome evolution.

### 3.3 Alignment to other kelp genomes

**Figure 2:**
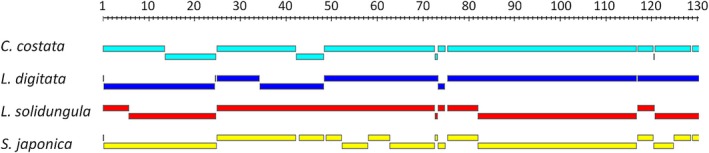
Synteny of the four kelp chloroplast genomes. The assembled genomes were mapped against the *Undaria. pinnatifida* genome using nucmer (Kurtz et al., 2004) and visualized with Bio:: Graphics (https://metacpan.org/release/LDS/Bio-Graphics-2.37). Colors for the different chloroplast genomes were chosen arbitrarily. The identity threshold for each segment was 90%, and small hits contained within a larger one were removed including the matches of the second repeat region. The scale represents the *U. pinnatifida* base positions in kb. The breaks indicate nucmer alignment breaks See Table A2. When gaps between alignments are small, the graphics software shifted the next alignment block to a lower position to emphasize the alignment gap positions

We then asked whether the whole chloroplast genomes were alignable, that is, are completely collinear between each other. To this end, we first made a nucmer alignment with the *U. pinnatifida* genome as reference, which showed that large segments of all chloroplast genomes could indeed be aligned (Figure 2). Only a few regions appear to be rearranged or contain larger insertions or deletions so that the similarity dropped below the 90% threshold. Missing or additional tRNAs are too small to cause such similarity breakpoints as the comparison of tRNA positions (Table A1) and nucmer similarity breakpoint positions shows (Table A2). We then aligned the chloroplast genomes with MAFFT which proved that the nucmer segments aligned in the same order in all chloroplast genomes and that therefore all kelp chloroplast genomes are collinear. However, closer inspection revealed that small rearrangements occurred involving the inverted repeat (IR) regions (Table 1). In comparison with *C. costata*, *S. japonica* and *U. pinnatifida* both *Laminaria* species have a gene directly adjacent of the IRs translocated to the other copy of the IR (Table 1). In *L. digitata rpl*21 is affected and in *L. solidungula ycf*37. Interestingly, *ycf*37 was presumably pseudogenized during this process in *L. solidungula* since the N terminal part of the protein is no longer encoded in this gene (Table A3).

**Table 1:** Chloroplast genome features of kelp species. The inverted repeat (IR) consists of the genes in the order 16S ribosomal RNA, tRNA‐Ile, tRNA‐Ala, 23S ribosomal RNA, 5S ribosomal RNA. The first row in each cell of the gene order column shows the neighboring genes of the forward repeat and the second row those of the reverse repeat for each species row

Species	Length (bp)	Inverted repeat length (bp)	Gene order found at boundaries of the two IR regions	Rearrangements
*Costaria costata*	129,947	5,409	rpl32‐tRNALeu‐**IR**‐rpl21‐rpl3 ycf17‐**IR**‐ycf37‐psaM	
*Laminaria digitata*	130,376	5,294	rpl32‐tRNALeu‐**IR**‐ rpl3 ycf17‐rpl21‐**IR**‐ycf37‐psaM	rpl21 at other IR
*Laminaria solidungula*	130,398	5,493	rpl32‐tRNALeu‐ycf37‐**IR**‐rpl21‐rpl3 ycf17‐**IR**‐psaM	ycf37 at other IR; pseudogene
*Saccharina japonica*	130,584	5,496	rpl32‐tRNALeu‐**IR**‐rpl21‐rpl3 ycf17‐**IR**‐ycf37‐psaM	
*Undaria pinnatifida*	130,383	5,404	rpl32‐tRNALeu‐**IR**‐rpl21‐rpl3 ycf17‐**IR**‐ycf37‐psaM	

### 3.4 Sequence variation across five chloroplast genomes

**Figure 3:**
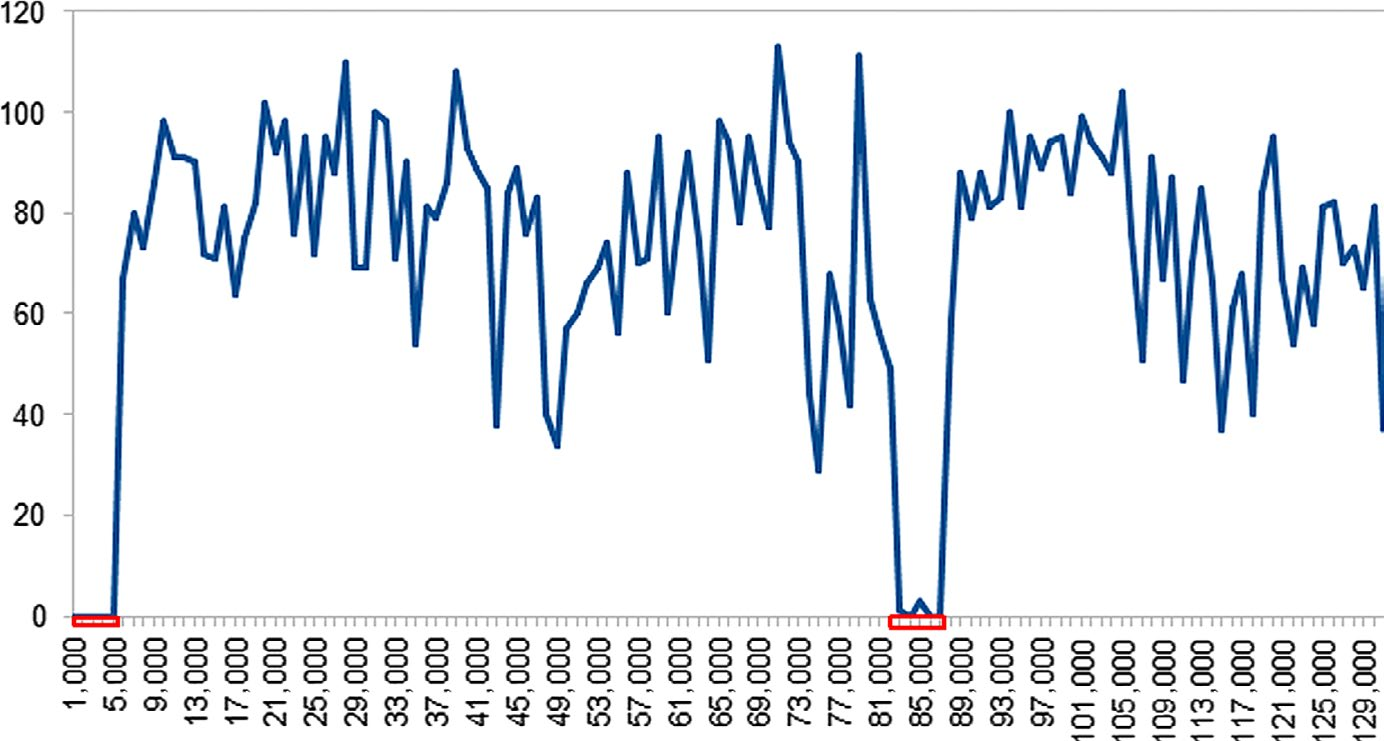
Single nucleotide polymorphism (SNPs) distribution over the kelp chloroplast genome. SNPs were detected by aligning short reads to the *Saccharina japonica* genome as a reference. All SNPs (see Table 2) from the aligned reads of the available four kelp species in windows of 1,000 bases were counted and plotted. X‐axis: Base count in the *S. japonica* reference. Y‐axis: number of SNPs. The red rectangles indicate the position of the inverted repeats

**Figure 4:**
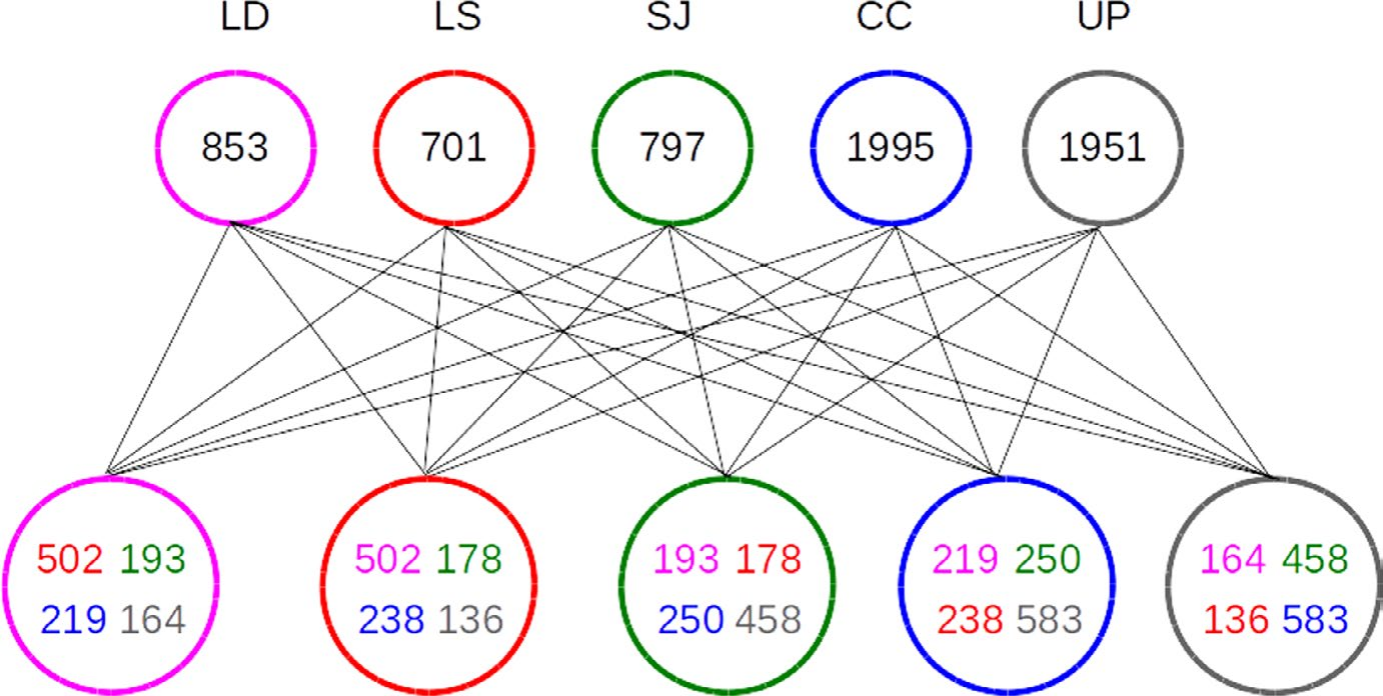
SNPs unique and shared between species. The upper circles show the unique SNPs in each species, and the lower row of circles indicates shared SNPs between two species with the numbers in the color of the respective species. To facilitate readability, circles are connected by lines. CC, *Costaria costata* (blue); LD, *Laminaria digitata* (magenta); LS, *L. solidungula* (red); SJ, *Saccharina japonica* (green); UP, *Undaria pinnatifida* (gray)

The collinearity of the chloroplast genomes allows alignment and definition of sequence variation irrespective of coding, noncoding, or intergenic regions. Since we, however, observed small rearrangements in the *Laminaria* species, we decided not to use the global alignment for single nucleotide polymorphism (SNP) and insertion or deletion (indel) detection. Instead, we analyzed the sequence variations locally using a 100× coverage of artificial reads each which we mapped to the *S. japonica* genome. In total, we found 9,218 SNPs and 164 indels. We counted all SNPs from all species in windows of 1,000 bases to examine the SNP distribution over the chloroplast genome (Figure 3). The SNPs are fairly equally distributed over the whole‐genome sequence, only the inverted repeat regions are nearly devoid of sequence variation. This phenomenon was already observed in higher plants (Zhu, Guo, Gupta, Fan, & Mower, 2016). By far, the highest numbers of unique SNPs are present in the genomes of *U. pinnatifida* and *C. costata* (Figure 4). Conversely, the *Laminaria* species have the largest set of SNPs in common (502) which likely evolved with the establishment of this lineage. Not surprisingly, the shared set of both *Laminaria* species with the most distantly related *U. pinnatifida* chloroplast genome is the smallest with 164 (*L. digitata*) and 136 (*L. solidungula*). The 583 SNPs shared between *C. costata* and *U. pinnatifida* likely represent the ancient state of the chloroplast genomes. The overall pattern of SNP evolution indicates that lineage and species‐specific SNPs accumulate over time as expected. However, SNPs were frequently observed to be scattered in the phylogeny indicating possible incomplete lineage sorting by, for example, recombination of heteroplasmic genomes.

Compared to SNPs indels are rare. In total, we detected 197 indels compared to the *S. japonica* genome. With 59 and 57, the number of indels in *C. costata* and *U. pinnatifida* is highest, whereas *L. solidungula* has only 29 indels and *L. digitata* 36. Indels can only be detected with our method if they are comparably small, that is, in the range of 10 bases. Larger indels exist as the similarity breaks indicate (Figure 2).

We then examined the ratio of SNPs between intergenic and genic (i.e., coding regions including RNA genes; Table 2). The ratio of genic to intergenic SNPs ranges from 15% to 19%. The number of detectable SNPs per kb is, however, slightly lower in intergenic compared to genic regions. Since most larger indels reside in the intergenic regions the alignability of these regions is reduced and thus the potential to detect SNPs. Overall, the number of SNPs per kb is comparable between intergenic and genic regions in all species (Table 2).

**Table 2:** Number of detected SNPs in genic and intergenic regions. *Saccharina. japonica* was used as a reference and artificially generated reads from the other kelp chloroplast genomes were mapped onto this reference (see section 2)

Total SNPs	Genome length (bp)	Genic regions (bp)	Intergenic regions (bp)	genic SNPs	Intergenic SNPs	Intergenic/genic	SNP/kb genic	SNP/kb intergenic
*Saccharina japonica*	130,584	108,847	21,737					
*Costaria costata*	129,947	108,550	21,397	3,615	688	0.2	33.3	32.2
*Undaria pinnatifida*	130,383	108,751	21,632	3,633	626	0.2	33.4	28.9
*Laminaria solidungula*	130,398	108,730	21,668	2,825	429	0.2	26.0	19.8
*Laminaria digitata*	130,376	108,647	21,729	2,961	566	0.2	27.3	26.0

The distribution of synonymous versus nonsynonymous SNPs in coding regions is also of interest (Table 3). For this analysis, we calculated for each species the number of SNPs in the two categories and tested, whether those SNPs also occurred in another species. As expected, nonsynonymous SNPs are much rarer than synonymous SNPs indicating purifying selection on the coding sequences. Some codons contain different SNPs in different species, resulting sometimes in the encoding of different amino acids. These 260 codons therefore seem to be less constrained in terms of exchangeability.

**Table 3:** Synonymous and nonsynonymous SNPs in coding regions. The table denotes SNPs occurring in single species versus all others and shared SNPs between two species. Different codon changes denote different nonsynonymous SNPs affecting the same codon, which lead to different amino acids in different species

SNP occurrence	All	Synonymous (s)	Nonsynonymous (*n*)	n/s %
*Saccharina japonica*	714	610	104	17.0
*Costaria costata*	1,596	1,448	148	10.2
*Undaria pinnatifida*	1,602	1,352	250	18.5
*Laminaria solidungula*	569	509	60	11.8
*Laminaria digitata*	672	600	72	12.0
*S. japonica* and *C. costata*	224	197	27	13.7
*S. japonica* and *U. pinnatifida*	404	390	14	3.6
*S. japonica* and *L. digitata*	159	151	8	5.3
*S. japonica* and *L. solidungula*	143	142	1	0.7
*C. costata* and *U. pinnatifida*	492	448	44	9.8
*C. costata* and *L. solidungula*	207	204	3	1.5
*C. costata* and *L. digitata*	177	162	15	9.3
*U. pinnatifida* and *L. digitata*	141	127	14	11.0
*U. pinnatifida* and *L. solidungula*	118	100	18	18.0
*L. digitata* and *L. solidungula*	432	391	41	10.5
Sum	7,650	6,831	819	12.0
Different codon changes			260	

The ratio of nonsynonymous to synonymous SNPs ranges from 10.2% to 18.5% in species and from 0.7% to 18% in species pairs. The partly lower values for species pairs might be caused by a lower likeliness of maintenance of nonsynonymous SNPs in two independent species. Interestingly, *S. japonica* and *U. pinnatifida* have the highest ratio of nonsynonymous to synonymous SNPs in their species specific SNPs, which could be due to a less efficient purifying selection or faster accumulation of mutations than in the other species. By calculating the d*N*/d*S* ration, we found no evidence for positive selection (i.e., d*N*/d*S* > 1) in any of the coding genes of the chloroplast genomes.

SNP pairs (i.e., mutations adjacent to each other or multinucleotide polymorphisms [MNPs]) are thought to be not always independent (Prendergast, Pugh, & Harris, 2018). We analyzed such pairs in the Kelp chloroplast genomes and found that they are generally rare, but are also partly shared between species (Table 4). Interestingly, these SNPs are equally distributed between genic and intergenic regions. Since intergenic regions cover a far smaller area of the chloroplast genome, the propensity for this kind of SNPs is to reside in intergenic regions.

**Table 4:** SNP pairs in kelp chloroplast genomes. Shared pairs between different species are also listed

	Genic	Intergenic
*Saccharina japonica*	7	0
*Costaria costata*	39	42
*Undaria pinnatifida*	31	31
*Laminaria solidungula*	10	11
*Laminaria digitata*	9	15
*S. japonica* and *C. costata*	0	0
*S. japonica* and *U. pinnatifida*	4	3
*S. japonica* and *L. digitata*	0	3
*S. japonica* and *L. solidungula*	2	0
*C. costata* and *U. pinnatifida*	5	3
*C. costata* and *L. solidungula*	7	1
*C. costata* and *L. digitata*	0	2
*U. pinnatifida* and *L. digitata*	0	0
*U. pinnatifida* and *L. solidungula*	1	0
*L. digitata* and *L. solidungula*	2	6
All	117	117

To exclude the possibility that population structure and sequence variation impact the SNP analyses, we retrieved *L. digitata* samples from 6 different locations (North Sea Heligoland, north east Atlantic Spitsbergen, northwest Atlantic Halifax, western Atlantic Connecticut, eastern Atlantic Roscoff, and Quiberon). We amplified a 850‐bp region containing the cbbx gene and part of the adjacent intergenic region from all samples, cloned the PCR products into vectors, and sequenced three clones each. We could not detect any variation indicating that variation of the chloroplast genome in the whole *L. digitata* population is rare. We then sequenced and assembled the complete plastid genomes from the Heligoland and Spitsbergen isolates and counted the differences to the reference sequence, which was derived from Heligoland. The chloroplast genome of the Spitsbergen isolate contained 27 SNPs and 9 small indels in total and the new Heligoland sample four SNPs and seven small indels, respectively. We therefore conclude that population variation does not impact our SNP analysis across species. Further studies are, however, needed to confirm the disruption of ycf37 in all individuals of *L. solidungula*.

## 4 DISCUSSION

The chloroplast genomes of photosynthetic eukaryotes are relatively stable and have a low substitution rate (Xu et al., 2015). We have analyzed two kelp species chloroplast genomes and compared them to available genomes of other kelp species. This analysis gives us deep insights into kelp evolution and may help to understand evolutionary processes in this phylogenetic branch.

### 4.1 Collinearity and stability of the chloroplast genomes

Only one or two tRNA genes are additionally inserted in the otherwise nearly collinear kelp chloroplast genomes. These additionally inserted tRNAs mainly have introns and are only a second copy of a tRNA species. Thus, these tRNAs would be dispensable and might occur and disappear frequently in evolution without affecting the collinearity. Only in the vicinity of the IRs, we observed translocations of genes in *Laminaria*. Such translocations could be connected to double strand break repair and homologous recombination at IR sites as it was also observed in higher plants (Zhu et al., 2016). The translocation of ycf37 in *L. solidungula* probably led to its defunctionalization since the N terminal part including the start codon of the gene is missing as the alignment indicates (Table A3). No start codon in the 5′ vicinity was found which could be used as alternative start from the ribosome. Further work will have to show whether or not a protein can be produced by this truncated gene locus. Functional analysis of a knockout mutant of ycf37 in *Synechococcus* revealed its involvement in the building of a specific photosystem I complex, which seems to be required under high light conditions (Dühring, Irrgang, Lünser, Kehr, & Wilde, 2006). It is possible that this protein is dispensable under the relatively lower light conditions in higher latitudes, for example (Pavlov *et al.*, in press), where *L. solidungula* thrives exclusively (Roleda, 2016).

### 4.2 SNP evolution

The evolutionary occurrence of the same mutation at a given location independently in different species is unlikely. Thus, if a SNP is found in two species, it should have the same origin, that is, one mutation event in the course of evolution. Our analysis shows that SNP presence and absence in kelp species chloroplast genomes does not follow the phylogeny; that is, we cannot trace back the first occurrence of a SNP in the phylogenetic tree. Thus, scattered occurrence of a SNP, for example, presence in *U. pinnatifida* and *L. solidungula* and absence in the other species does not mean that this SNP was lost in these lineages independently. Rather, this scattered occurrence can most easily be explained by the presence of heteroplasmic chloroplast genomes with homologous recombination between them. Thus, our study reveals for the first time incomplete lineage sorting in kelp species as it was shown in higher plants (Jakob & Blattner 2006; Sabir et al., 2014). The amount of SNPs per kb cannot be used to discern between coding and noncoding regions, since they are nearly equally distributed over the whole chloroplast genomes. This equal distribution of SNPs over the whole chloroplast genome except the inverted repeat regions can be due to equal constraints on intergenic and genic regions, if we assume saturation with mutations. This would imply that regulatory or other functions are encoded in the intergenic regions. The occurrence of multinucleotide mutational events seems to be triggered by infidelities of the DNA polymerase (Schrider, Hourmozdi, & Hahn, 2011; Venkat, Hahn, & Thornton, 2018). Here, we could show that such substitutions are rarer in coding sequences than in intergenic regions. The lower amount of multinucleotide mutations per kb in genic regions of the chloroplast genomes is likely due to purifying selection. We observed a variation of Kelp chloroplast genomes in pairwise comparisons of 2.5%–3.3%. For Gossypium (cotton) species, the variation was determined to be at 0.6% (Xu et al., 2012) with a divergence time of roughly 12.5 mya (Wendel et al., 2010). For Oryza (rice), the variation is 0.36% (Wambugu, Brozynska, Furtado, Waters, & Henry, 2015) with a divergence time of Oryza estimated to be at around 10 mya (Kellogg, 2009). The first Kelp forests occurred in the Miocene around 22 mya together with grass lands. Thus, their evolution started much earlier than the establishment of either rice or cotton families. We therefore Kelp chloroplast genomes seem to evolve at comparable rates as land plant families.

## 5 CONCLUSION

Our analysis of kelp chloroplast genomes broadens our view on the evolution of these important species. It is possible that either the pseudogenization of the chloroplast gene ycf37 led to the adaptation and confinement of *L. solidungula* to the Arctic, or the low light habitat choice made ycf37 dispensable. The analysis of SNP distribution shows that no positive selection acts on coding sequences in kelp chloroplast genomes. Rather, the relative scarcity of multinucleotide substitutions in genic regions compared to nongenic regions shows that purifying selection is at work in genic regions. Thus, not only single SNPs should be taken into account before far reaching conclusions on chloroplast genome evolution can be drawn.

## AUTHOR CONTRIBUTIONS

GG conceived the study and wrote the manuscript. SR carried out the experiments and analyzed the data. IB contributed material and to the final version of the manuscript. KV contributed to the discussions of the results. All authors provided critical feedback.

## Figures and Tables

**Table A3:**
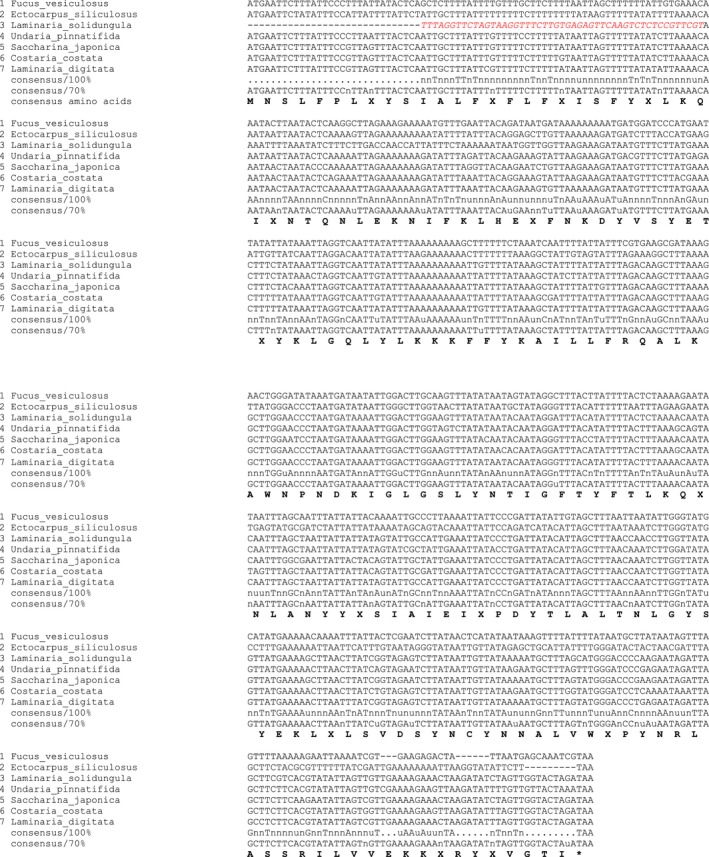
Alignment of chloroplast gene ycf37 from different brown algae. The nonhomologous sequence part of *Laminaria solidungula* is colored in red. The amino acid translation was made from the 70% consensus sequence, where a nucleotide was taken if at least seventy percent of the aligned sequences have the same letter. n in consensus denotes any nucleotide at that alignment position, and u stands for purin bases at that position

## Data Availability

The annotated chloroplast genomes are available under the accession numbers MH784527 (*L. solidungula*) and MH784528 (*L. digitata*).

## APPENDIX 1

**Table A1:** tRNAs and their positions in the Kelp genomes. CC, *Costaria costa*ta; LD, *Laminaria digitata*; LS, *L. solidungula*; SJ, *Saccharina japonica*; UP, *Undaria pinnitafida*

Number of tRNAs	31		28		31		29		30	
Species	UP		CC		SJ		LD		LS	
tRNA	start	stop	start	stop	start	stop	start	stop	start	stop
Met					5,832	5,899				
Phe	7,288	7,360	7,293	7,365	7,148	7,220	7,126	7,198	7,143	7,215
Tyr	27,354	27,434	27,433	27,513	27,249	27,329	27,279	27,359	27,236	27,316
Sup (intron)	34,028	34,266							33,945	34,190
Asp	38,590	38,664	38,727	38,801	38,498	38,572	38,600	38,674	38,529	38,603
Ile (intron)	50,988	51,192								
Arg	51,998	52,070	52,161	52,233	51,870	51,942	51,929	52,001	51,872	51,944
Glu	52,127	52,199	52,290	52,362	51,999	52,071	52,058	52,130	52,001	52,073
Ile (intron)	59,171	59,416								
Leu	81,865	81,946	81,703	81,784	81,909	81,990	81,785	81,866	81,854	81,935
Ile	83,943	84,016	83,860	83,933	84,124	84,197	83,915	83,988	84,195	84,268
Ala	84,020	84,092	83,937	84,009	84,201	84,273	83,992	84,064	84,272	84,344
Gly (intron)			96,734	96,976						
His	116,468	116,540	116,392	116,463	116,650	116,722	116,377	116,449	116,776	116,848
Thr	116,618	116,690	116,545	116,617	116,804	116,876	116,531	116,603	116,930	117,002
Val	117,062	117,133	116,874	116,945	117,160	117,231	116,914	116,985	117,317	117,388
Arg	117,156	117,228			117,254	117,326	117,008	117,080	117,411	117,483
Phe (intron)					117,852	117,957				
Asn	120,522	120,593	120,233	120,304	120,563	120,634	120,381	120,452	120,789	120,860
Arg	128,196	128,269	127,843	127,916	128,307	128,380	128,066	128,139	128,471	128,544
Gln	128,311	128,382	127,951	128,022	128,414	128,485	128,181	128,252	128,591	128,662
Leu (intron)							116,823	116,617		
Trp	109,963	109,891	109,885	109,813	110,141	110,069	109,912	109,840	110,272	110,200
Gly	78,187	78,117	78,029	77,959	78,220	78,150	78,094	78,024	78,169	78,099
Lys	72,723	72,652	72,767	72,696	72,690	72,619	72,778	72,707	72,697	72,626
Cys	49,214	49,144	49,362	49,292	49,084	49,014	49,174	49,104	49,106	49,036
Lys (intron)					43,701	43,498			43,708	43,505
Met	43,556	43,471	43,710	43,625	43,453	43,368	43,562	43,477	43,461	43,376
Met	38,427	38,355	38,569	38,497	38,339	38,267	38,441	38,369	38,371	38,299
Ser	38,347	38,258	38,489	38,400	38,259	38,170	38,361	38,272	38,291	38,202
Gly	35,888	35,817	35,999	35,928	35,770	35,699	35,865	35,794	35,798	35,727
Pro	25,512	25,439	25,613	25,540	25,430	25,357	25,458	25,385	25,416	25,343
Met	25,383	25,310	25,492	25,419	25,309	25,236	25,337	25,264	25,295	25,222
Ser	24,675	24,588	24,754	24,667	24,564	24,477	24,588	24,501	24,575	24,488
Ile	3,464	3,391	3,466	3,393	3,286	3,213	3,288	3,215	3,287	3,214
Ala	3,387	3,315	3,389	3,317	3,209	3,137	3,211	3,139	3,210	3,138

**Table A2:** Nucmer segments of Kelp genomes mapped to the *U. pinnitafida* genome (corresponds to Figure 2)

CC	Costaria	Segments	9	13,403	.	+	1
CC	Costaria	Segments	13,533	24,692	.	+	1
CC	Costaria	Segments	24,859	42,156	.	+	1
CC	Costaria	Segments	42,290	48,198	.	+	1
CC	Costaria	Segments	48,384	72,508	.	+	1
CC	Costaria	Segments	72,648	73,200	.	+	1
CC	Costaria	Segments	73,336	74,800	.	+	1
CC	Costaria	Segments	75,314	116,698	.	+	1
CC	Costaria	Segments	116,987	120,312	.	+	1
CC	Costaria	Segments	120,439	120,642	.	+	1
CC	Costaria	Segments	120,746	128,507	.	+	1
CC	Costaria	Segments	128,991	130,383	.	+	1
SJ	Saccharina	Segments	1	177	.	+	1
SJ	Saccharina	Segments	178	24,745	.	+	1
SJ	Saccharina	Segments	24,854	42,083	.	+	1
SJ	Saccharina	Segments	42,946	48,206	.	+	1
SJ	Saccharina	Segments	48,778	52,199	.	+	1
SJ	Saccharina	Segments	52,297	57,837	.	+	1
SJ	Saccharina	Segments	58,025	62,661	.	+	1
SJ	Saccharina	Segments	62,748	72,506	.	+	1
SJ	Saccharina	Segments	72,643	73,187	.	+	1
SJ	Saccharina	Segments	73,341	74,776	.	+	1
SJ	Saccharina	Segments	75,310	81,960	.	+	1
SJ	Saccharina	Segments	82,003	116,698	.	+	1
SJ	Saccharina	Segments	116,995	120,286	.	+	1
SJ	Saccharina	Segments	120,474	124,772	.	+	1
SJ	Saccharina	Segments	124,883	128,515	.	+	1
SJ	Saccharina	Segments	128,991	130,383	.	+	1
LD	L_digitata	Segments	1	177	.	+	1
LD	L_digitata	Segments	178	24,331	.	+	1
LD	L_digitata	Segments	24,575	24,692	.	+	1
LD	L_digitata	Segments	24,853	34,190	.	+	1
LD	L_digitata	Segments	34,292	48,199	.	+	1
LD	L_digitata	Segments	48,416	73,211	.	+	1
LD	L_digitata	Segments	73,341	74,756	.	+	1
LD	L_digitata	Segments	75,313	116,698	.	+	1
LD	L_digitata	Segments	116,971	130,383	.	+	1
LS	L_solidungula	Segments	5,639	24,692	.	+	1
LS	L_solidungula	Segments	5,669	1	.	+	1
LS	L_solidungula	Segments	24,853	72,534	.	+	1
LS	L_solidungula	Segments	72,644	73,206	.	+	1
LS	L_solidungula	Segments	73,336	74,760	.	+	1
LS	L_solidungula	Segments	75,313	81,953	.	+	1
LS	L_solidungula	Segments	82,003	116,697	.	+	1
LS	L_solidungula	Segments	116,971	120,642	.	+	1
LS	L_solidungula	Segments	120,746	130,383	.	+	1
